# Intensified Use of Reproductive Technologies and Reduced Dimensions of Breeding Schemes Put Genetic Diversity at Risk in Dairy Cattle Breeds

**DOI:** 10.3390/ani10101903

**Published:** 2020-10-17

**Authors:** Anna-Charlotte Doublet, Gwendal Restoux, Sébastien Fritz, Laura Balberini, Guillaume Fayolle, Chris Hozé, Denis Laloë, Pascal Croiseau

**Affiliations:** 1 ALLICE, 149 rue de Bercy, 75012 Paris, France; anna-charlotte.doublet@inrae.fr (A.-C.D.); sebastien.fritz@allice.fr (S.F.); chris.hoze@allice.fr (C.H.); 2 Université Paris-Saclay, INRAE, AgroParisTech, GABI, 78350 Jouy-en-Josas, France; gwendal.restoux@inrae.fr (G.R.); denis.laloe@inrae.fr (D.L.); 3 AURIVA-Elevage, 61 Chemin des Hoteaux, 69126 Brindas, France; laura.balberini@auriva-elevage.fr; 4 Groupe UMOTEST, Les Soudanières, 01250 Ceyzériat, France; gfayolle@umotest.com

**Keywords:** inbreeding, MOET, OPU-IVF, simulation study, genomic selection

## Abstract

**Simple Summary:**

Maintaining genetic diversity in dairy cattle breeds is essential to support good performance and avoid inbreeding depression. This diversity could be threatened, however, by the recent increase in the use of reproductive technologies and the limited number of bulls in dairy cattle breeds. This study aimed to investigate the effects of these practices by simulating 15 breeding schemes similar to those carried out in the main dairy cattle breeds in France. We found that intensive use of reproductive technologies resulted in improved genetic gain, but diminished genetic diversity. However, this detrimental effect could be mitigated by maintaining a sufficient number of bulls in the breeding scheme.

**Abstract:**

In the management of dairy cattle breeds, two recent trends have arisen that pose potential threats to genetic diversity: the use of reproductive technologies (RT) and a reduction in the number of bulls in breeding schemes. The expected outcome of these changes, in terms of both genetic gain and genetic diversity, is not trivial to predict. Here, we simulated 15 breeding schemes similar to those carried out in large French dairy cattle breeds; breeding schemes differed with respect to their dimensions, the intensity of RT use, and the type of RT involved. We found that intensive use of RT resulted in improved genetic gain, but deteriorated genetic diversity. Specifically, a reduction in the interval between generations through the use of ovum pick-up and in vitro fertilization (OPU-IVF) resulted in a large increase in the inbreeding rate both per year and per generation, suggesting that OPU-IVF could have severe adverse effects on genetic diversity. To achieve a given level of genetic gain, the scenarios that best maintained genetic diversity were those with a higher number of sires/bulls and a medium intensity of RT use or those with a higher number of female donors to compensate for the increased intensity of RT.

## 1. Introduction

The degree of inbreeding and, more generally, the loss of genetic diversity in domestic animal populations have been the subject of particular attention since the first half of the last century [1]. Low genetic diversity leads to lower expected genetic gain [2,3], altered adaptive potential, and increased inbreeding depression, which all have a direct cost for breeders and breeding companies [4,5]. Human-designed breeding schemes may play a key role as strong selection in domestic populations may result in low genetic diversity [6]. This is especially true in dairy cattle breeds, for which the management of genetic diversity has become an important challenge.

One of the main approaches to managing genetic diversity in a dairy cattle breed is through breeding schemes, particularly with respect to the number of animals considered at each of the selection steps [7,8]. One important factor in the design of breeding schemes is the number of sires of bulls and marketed bulls included in the scheme. In addition, breeders must determine if they want to use reproductive technologies (RT hereafter), and if so, they must make decisions regarding the type and intensity of RT and the number of females to be used as embryo or oocyte donors [8,9,10].

In dairy cattle breeds, breeding schemes are designed and implemented by breeding companies with the goal of producing elite bulls whose high-genetic-quality semen can be commercialized. The use of artificial insemination is widespread, with the result that genetic diversity in a breed is highly correlated with genetic diversity in breeding schemes [11]. Currently, genetic diversity in French breeding programs is mostly managed by controlling the number of half or full siblings chosen as male candidates for selection, but kinship ties further back in the pedigree tend to be largely ignored [12].

Genetic diversity in French dairy cattle breeds is typically monitored using pedigree-based inbreeding estimates (*VARiabilité génétique des RUMinants et des Equidés*, genetic variability in ruminants and equines, VARUME) [13]. However, more accurate and comprehensive evaluations can be obtained using genomic data [6,14,15]. In particular, inbreeding estimates based on genotypes and runs of homozygosity (ROH), in particular, have been shown to be as efficient, if not more, in evaluating and managing genetic diversity [16]. Although pedigree information is still very useful, genomic or ROH-based inbreeding estimates can provide more detailed data for better monitoring of genetic diversity.

The aim of a breeding company is to increase the genetic gain. This is generally achieved by an increase in selection intensity using RT, which is accompanied by a decrease in the number of bulls considered and the interval between generations. The use of RT drastically modifies breeding schemes and can have significant impacts on genetic diversity. The same can also be said of other technologies such as genomic selection (GS). On one hand, it is possible to use GS to preserve genetic diversity (e.g., by increasing the number of candidates for selection and incorporating a wider range of bulls). On the other, however, the use of GS can also pose a threat to genetic diversity (due to shorter generation intervals, for example). Some studies have shown that the implementation of GS programs resulted in an accelerated loss of genetic diversity, with severe increases in inbreeding rates (both per year and per generation) in breeds such as Holstein [7,17,18]. However, this loss was not observed in other breeds because of differences in the breeding schemes and the ways bulls are chosen. For instance, in France, Montbéliarde and Normande breeds were able to maintain their inbreeding rates while increasing their genetic gain [7]. Thus, the beneficial or detrimental effects of such technologies depend on the breeding scheme(s) and the population in question. The management of breeding schemes therefore represents a key factor in the management of genetic diversity and genetic gain in dairy cattle breeds.

In large dairy cattle breeds, RTs such as multiple ovulation and embryo transfer (MOET) and ovum pick-up and in vitro fertilization (OPU-IVF) are commonly used. In combination with genomic selection, RT appears to be very beneficial for genetic gain in dairy breeds [19,20,21,22,23]. The increased genetic gain associated with RT is due to an intensification in the use of the best females, with higher selection intensity [24], and to a reduction in the intervals between generations. With OPU-IVF, generation intervals can be shortened even more, as OPU can be performed on immature young heifers as soon as two months of age [25]. Taken together, these effects tend to increase the loss of genetic diversity, but this can be at least partially counterbalanced with the use of genomic selection to more-accurately evaluate candidates for selection and thus reduce within-family selection [15]. Moreover, the true effects of the different types of RT on genetic gain and genetic diversity depend on the number of female donors in the breeding scheme and the intensity of their use (number of calves born from RT) [10]. This is also true for the selection of bulls. The cost of breeding one bull has decreased dramatically since the implementation of genomic selection, as this process has nearly put an end to expensive progeny testing (€45,000 on average per bull in France, although this value varies depending on the breed). Reducing the number of bulls also reduces the total cost of breeding bulls, allowing breeding companies to invest more money in RT, for example. The complexity of the system, and the number of factors involved, can make it difficult to determine the outcome of these types of choices with respect to their effects on genetic gain and genetic diversity.

The objective of this study was, in the context of genomic selection of dairy cattle breeds, to quantify the genetic gain and the loss of genetic diversity associated with different breeding schemes. Our aim was to provide useful recommendations to breeding companies to help with the overall management of breeding programs and, specifically, with the implementation of RT (MOET or OPU-IVF). We simulated 15 genomic selection breeding schemes that differed in dimensions (numbers of sires of bulls, marketed bulls, and female donors), the intensity of RT use, and the type of RT involved. These scenarios were then evaluated in terms of genetic gain and ROH-based inbreeding rate using stochastic simulations.

## 2. Materials and Methods

### 2.1. Simulated Population and Scenarios

Multiple scenarios were designed that differed in the extent of the use of embryo transfer and that relied on different numbers of sires of bulls and marketed bulls. These were tested by stochastic simulations of a breeding program that was similar in dimensions to those carried out in large French dairy cattle breeds.

Stochastic simulations were conducted using the MoBPS R package, version 1.0.2 (Göttigen, Germany) [26,27]. We simulated the same number of genetic markers as are present on the publicly available medium-density SNP chip (Illumina Infinium^®^ BovineSNP50 BeadChip, San Diego, CA, USA), which is widely used in genomic selection programs around the world. We only considered the 29 bovine autosomes, on which we analyzed 41,377 SNP markers that were distributed regularly with respect to recombination rates (i.e., the distances in centimorgans between neighboring SNPs were approximately equal) [28]. On average, one SNP was simulated every 60.4 kb. We simulated 500 additive quantitative trait loci (QTLs), randomly distributed along the genome, whose effects were drawn from a gamma distribution (shape = 0.4 and scale = 5) [29] (see Supplementary Table S1).

Animals were selected according to the breeding goal, which can mimic either a single trait or a multi-trait synthetic index (e.g., [30]); the latter consists of an estimated breeding value (EBV) based on the true breeding values generated by the simulation of genotypes. The gross true breeding value for individual *i* (gTBV*_i_*) was calculated as the sum of all additive effects of QTLs of individual *i* (see Table S1). We mimicked genomic evaluations with a constant coefficient of determination (*CD*) of 0.7.

Assuming a constant CD for each individual, the joint distribution of EBV*_i_* and gTBV*_i_* for an individual *i* is [31]:

$\left(\begin{array}{c} gTBV_{i}\\ EBV_{i} \end{array}\right)\sim N_{2}\left[\left(\begin{array}{c} 0\\ 0 \end{array}\right),\left(\begin{array}{cc} \sigma_{a}^{2} & CD\sigma_{a}^{2}\\ CD\sigma_{a}^{2} & CD\sigma_{a}^{2} \end{array}\right)\right]$, where $\sigma_{a}^{2}$ is the additive genetic variance (or the variance of gTBV in the founding population).

Knowing gTBV*_i_*, the conditional distribution of EBV*_i_* is then [32]:

(1) $$EBV_{i}|gTBV_{i}\;\sim N(CD\times gTBV_{i},\left(1-CD\right)CD\sigma_{a}^{2})$$

For each individual *i*, EBV*_i_* was drawn once from this normal distribution. Genotypes were used for the evaluation of ROH-based inbreeding.

Before we applied the different scenarios, we instituted a burn-in process: the objective was to obtain 10 simulated populations (one for each of the 10 replicates for each scenario) with ROH-based inbreeding around 10–11%, similar to what is observed in real French dairy cattle breeds [7]. For this, we simulated 10 founding populations of 100 cows and 10 bulls and performed selection on EBV until the size (20,000 elite cows and heifers spread over four consecutive birth years) and inbreeding of the simulated populations reached our goal values. This generated linkage disequilibrium and genomic structure in the simulated populations.

**Figure 1:**
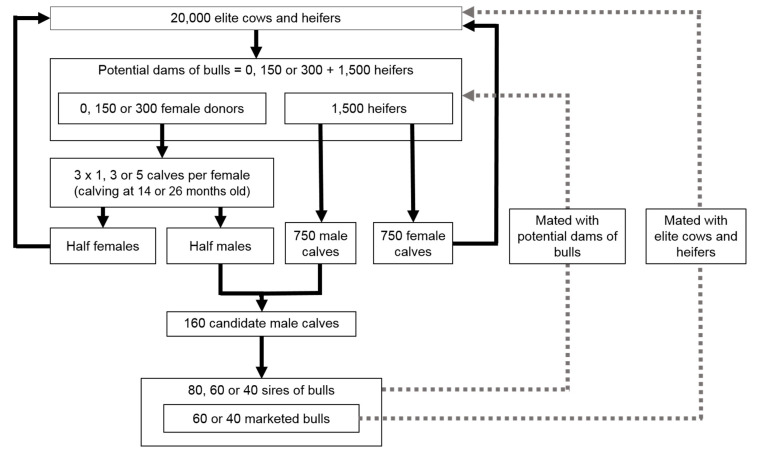
Selection steps in the simulated dairy cattle breeding scheme using embryo transfer.

Next, we simulated 10 replicates for each of the 15 different scenarios of genomic selection schemes based on embryo transfer, conducted for 20 years. Selection was performed using genomic evaluation for both young bulls and potential dams of bulls (see Figure 1 and Table 1). The 15 scenarios differed in their dimensions, intensity of RT use, and the type of RT applied.

Each simulated population consisted of 20,000 elite cows and heifers spread over four consecutive birth years, obtained at the end of the burn-in process described above. Only heifers could be selected as potential dams of bulls. In the reference scenario (REF), no RT was used. In all other scenarios, the 150 or 300 heifers with the highest estimated breeding values (EBV) were chosen to be embryo donors. We compared different intensities in the use of embryo transfer: low-intensity scenarios (three calves per female donor), medium-intensity scenarios (nine calves per female donor), and high-intensity scenarios (15 calves per female donor). In the context of high-intensity RT use, two types of RT were compared: MOET-like scenarios (five bulls used to obtain 15 calves) and OPU-IVF-like scenarios (15 bulls used to obtain 15 calves), with either medium or short generation intervals between female donors (calving at either 26 or 14 months, respectively). In most cases, female donors were mated randomly with 80 sires of bulls. We also simulated high-intensity scenarios with a medium or low number of sires of bulls (60 or 40 sires, respectively) to estimate the effect of a reduction in the number of sires. The resulting 1500 heifers with the highest EBV were randomly mated with the same number of sires of bulls by conventional insemination.

In all but three scenarios (H, I, N; see Table 1), all heifers and cows not selected as potential dams of bulls were randomly mated with 60 marketed bulls, chosen from the sires of bulls with the highest EBV. In high-intensity scenarios, we also estimated the effect of decreasing the number of marketed bulls by simulating scenarios with a low number of marketed bulls (40 bulls) (scenarios H, N, and I; see Table 1). Each cow reproduced three years in a row and obtained one calf per reproductive year (except when they were chosen as female donors). The cow population was renewed by keeping heifers with the highest EBV for replacement.

Sires of bulls were selected randomly from the 160 male calves with highest EBV born from the potential dams of bulls. The sires of bulls with the highest EBV became marketed bulls. These young sires and bulls were two years old when their calves were born and used only one year.

A bull (or cow) could not have more than five daughters (three daughters, respectively) chosen as female donors and five sons (three sons, respectively) chosen as male candidates for selection. All sires had at least one female offspring chosen as a female donor and one male offspring chosen as a candidate for selection. These constraints were based on current practices in large French dairy cattle breeding schemes.

### 2.2. Evaluation of Genetic Gain and Genetic Diversity Outcomes

Since different genetic gains could be expected between the sexes, we evaluated the outcomes of each scenario in terms of genetic gain and genetic diversity in two distinct subpopulations: (i) cows, defined as all females with at least one calf; and (ii) sires and bulls, defined as all males that became sires of bulls and/or marketed bulls.

Genetic gain was evaluated based on the evolution of the true breeding value TBV*_i_*, expressed as:

(2) $${\mathrm{TBV}}_{i}=\frac{{\mathrm{gTBV}}_{i}-mean\left({\mathrm{gTBV}}_{year\;1}\right)}{\sigma \left({\mathrm{gTBV}}_{year\;1}\right)}$$

with mean(gTBV*_year 1_*) the mean and *σ*(gTBV*_year 1_*) the standard derivation of gTBV for individuals born in the first year after implementation of the scenario. As we relied on TBV, the Bulmer effect was taken into account in this estimation of genetic gain.

Genetic diversity was evaluated based on inbreeding estimated from runs of homozygosity (ROH-based inbreeding) [33]. ROHs correspond to autozygous portions of the genome [33,34]. A ROH was defined as a homozygous segment with a minimum length of at least 15 SNPs or 1000 kb, with a minimum density of one SNP per 1000 kb. Two consecutive SNPs could not be included in the same ROH if they were over 1000 kb apart. We detected ROHs using the “homozyg” function of PLINK 1.9 [35,36] (command line: plink --cow --bfile genotyping_data_filename --homozyg --homozyg-kb 1000 --homozyg-snp 15 --homozyg-window-snp 15 --homozyg-density 1000 --out output_filename).

ROH-based inbreeding estimates, *F*_ROH,*i*_, were computed as follows [33]:

(3) $$F_{\mathrm{ROH},i}=\frac{\Sigma L_{\mathrm{ROH},i}}{L_{\mathrm{auto}}}$$

with $\Sigma L_{\mathrm{ROH},i}$ the total length of ROHs for animal *i*, and $L_{\mathrm{auto}}$ the length of the autosomal genome covered by SNPs, after removing gaps of more than 1000 kb between two SNPs. ROH-based inbreeding estimates were expressed in percentages.

Both annual genetic gain (based on TBV) and annual ROH-based inbreeding rate were modeled using the following linear model:

(4) $$Y_{ijk}=\beta_{j}^{\left[1\right]}+\beta_{k|j}^{\left[2\right]}+\alpha_{j}\times {\mathrm{Year}}_{i}+\epsilon_{ijk}$$

with *Y_ijk_* the variable of interest (TBV or ROH-based inbreeding) for individual *i* in the *k*th replicate of scenario *j* and born in the year Year*_i_*, $\beta_{j}^{\left[1\right]}$ the intercept of the model for scenario *j*, and $\beta_{k|j}^{\left[2\right]}$ the intercept of the model for the *k*th replicate of scenario *j*. *α* is the regression coefficient of the model, corresponding to the slope of the model; it represents the annual trend of either genetic gain or genomic inbreeding. All computations were performed using the “lm” function of R [37]. Slopes were compared using the “emtrends” function from the emmeans package [38] and the “cld” function of the multcomp package [39].

To facilitate comparison between scenarios, these slopes were transformed with respect to the reference scenario, REF (see Table 1), in other words, we calculated the ratio between the estimated value of the parameter for a given scenario and the estimated value of the parameter for the reference scenario (no use of embryo transfer).

## 3. Results

### 3.1. General Observations

The annual ROH-based inbreeding rate (∆*F*_ROH_) for the reference scenario (no use of RT) was 0.095% in cows (see Table 2) and 0.047% in sires and bulls (see Table 3) (0.235% and 0.094% per generation, respectively; see Supplementary Table S2). The annual genetic gain in TBV (∆*G*_TBV_) for the reference scenario was 0.298 in cows and 0.280 in sires and bulls (see Table 2 and Table 3). The ∆*F*_ROH_ for cows was thus higher by a factor of 2.02 than that of sires and bulls, while ∆*G*_TBV_ values for cows and for sires and bulls were more similar (∆*G*_TBV_ of cows was slightly higher, by a factor of 1.06). The smallest values of ∆*F*_ROH_ and ∆*G*_TBV_ were always observed when no RT was used (scenario REF), for cows as well as for sires and bulls.

The highest values of ∆*F*_ROH_, for both cows and sires/bulls, were observed in the high-intensity OPU-IVF-like scenario with short generation intervals and low numbers of sires and bulls, with ∆*F*_ROH_ = 0.219% and 0.220%, respectively (scenario N; see Table 2 and Table 3) (0.501% and 0.356% per generation, respectively; see Table S2). The highest values of ∆*G*_TBV_ for both cows and sires/bulls were observed in the high-intensity OPU-IVF-like, short interval scenario, with ∆*G*_TBV_ = 0.415 and 0.405, respectively (scenario M, see Table 2 and Table 3).

The comparison of ∆*F*_ROH_ from the different scenarios with that of scenario REF (∆*F*_ROH_/REF) ranged between 1.05 and 2.31 for cows, and between 1.05 and 4.67 for sires and bulls (see Table 3). The comparison of ∆*G*_TBV_ from the different scenarios with that of scenario REF (∆*G*_TBV_/REF) ranged between 1.08 and 1.48 for cows, and between 1.08 and 1.45 for sires and bulls (see Table 3).

**Figure 2:**
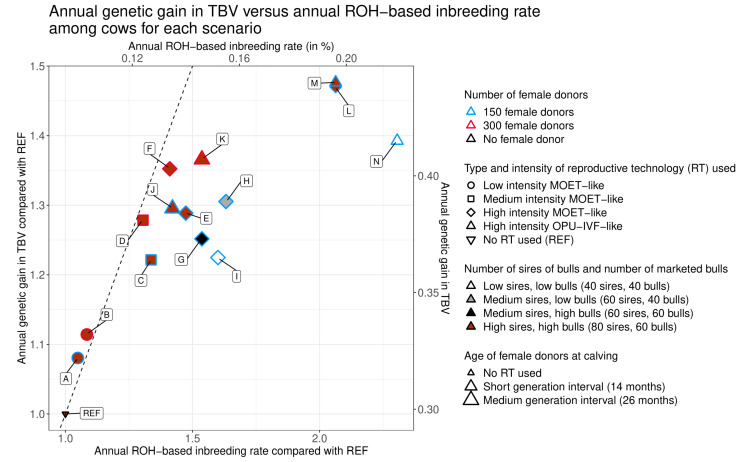
Annual genetic gain versus annual ROH-based inbreeding rate among cows. Letters indicate the names of the tested scenarios. Comparisons with REF were calculated as the ratio between the estimated value of the parameter for a given scenario and the estimated value of the parameter for the reference scenario (no use of embryo transfer). The dotted line is the line of identity between the annual ROH-based inbreeding rate compared with REF and the estimated annual genetic gain in true breeding value compared with REF.

**Figure 3:**
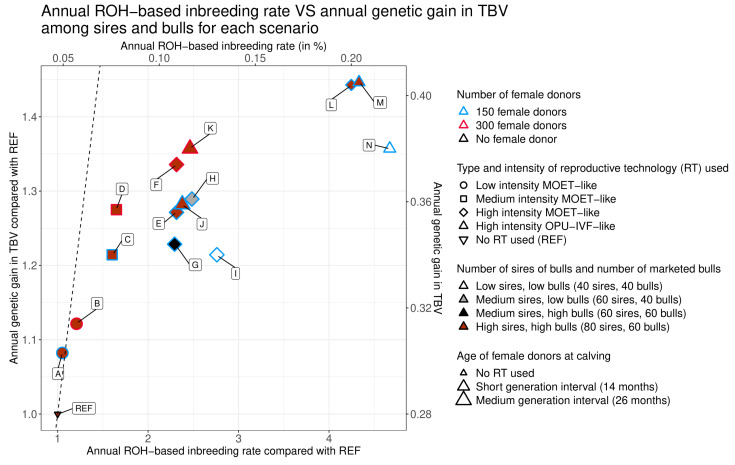
Annual genetic gain versus annual ROH-based inbreeding rate among sires and bulls. Letters indicate the names of the tested scenarios. Comparisons with REF were calculated as the ratio between the estimated value of the parameter for a given scenario and the estimated value of the parameter for the reference scenario (no use of embryo transfer). The dotted line is the line of identity between the annual ROH-based inbreeding rate compared with REF and the estimated annual genetic gain in true breeding value compared with REF.

In Figure 2 and Figure 3, for a given genetic gain (∆*G*_TBV_), the scenarios further to the left were those with the lowest inbreeding rates (∆*F*_ROH_); these are therefore the scenarios that offer the best compromise between genetic gain and genetic diversity. For instance, if we consider three clusters with different values of ∆*G*_TBV_/REF (between 1.2 and 1.25; between 1.25 and 1.3; between 1.35 and 1.4), the scenarios that best balance genetic gain and inbreeding rate within each cluster were C (medium intensity), D (medium intensity 300), and F (high intensity 300 MOET-like), respectively (see Figure 2 and Figure 3).

### 3.2. Intensity of Reproductive Technologies (RT) Use and Number of Female Donors

For cows, all scenarios with female donors and embryo transfer had values of ∆*F*_ROH_ and ∆*G*_TBV_ that were significantly higher than those of the scenario with no RT (scenario REF; see Table 2 and Table 3 and Figure 2 and Figure 3). For sires and bulls, the difference was significant only for ∆*G*_TBV_. Even at a low intensity of RT use and with 150 female donors, the inbreeding rate increased by a factor of 1.05 and the genetic gain for both cows and sires/bulls increased by a factor of 1.08 (scenario A; see Table 2 and Table 3).

Regardless of whether 150 or 300 female donors were used, the more intensive the use of RT (from scenarios A to F), the higher ∆*F*_ROH_ and ∆*G*_TBV_ were, with all scenarios being significantly different from one another (see Table 2 and Table 3 and Figure 2 and Figure 3). However, with an increase in the intensity of RT, values of ∆*F*_ROH_/REF increased more than ∆*G*_TBV_/REF. In the high-intensity MOET-like scenario with 150 female donors (scenario E), these values reached, respectively, 1.47 and 1.29 for cows and 2.31 and 1.27 for sires and bulls, while in the same RT scheme with 300 donors (scenario F), ∆*F*_ROH_/REF and ∆*G*_TBV_/REF were 1.41 and 1.35 for cows and 2.31 and 1.34 for sires and bulls, respectively (see Table 2 and Table 3).

All other parameters being equal, the scenarios with 300 female donors (scenarios B, D, F, and K; see Table 2 and Table 3) always achieved significantly higher values of ∆*G*_TBV_ than scenarios with 150 female donors (scenarios A, C, E, and J; see Table 2 and Table 3). However, this was not true for ∆*F*_ROH_, which, depending on the comparison, was higher, lower, or not significantly different between scenarios with 300 female donors (scenarios B, D, F, and K; see Table 2 and Table 3) and scenarios with 150 female donors (scenarios A, C, E, and J; see Table 2 and Table 3).

### 3.3. Generation Interval between Female Donors and Type of RT Used

The differences between MOET and OPU-IVF are that (i) OPU-IVF can be performed earlier in the life of the heifer than MOET, leading to shorter generation intervals between female donors (14 months instead of 26), and (ii) OPU-IVF allows each oocyte to be fertilized by a different sire, whereas MOET only permits fertilization by one sire per flushing. To compare the use of MOET and OPU-IVF, we simulated high-intensity MOET-like and OPU-IVF-like scenarios.

#### 3.3.1. Generation Interval between Female Donors

We first estimated the impact of generation intervals between female donors by comparing high-intensity scenarios that had either medium (calving at 26 months) or short generation intervals (calving at 14 months). For high-intensity MOET-like scenarios, ∆*F*_ROH_ and ∆*G*_TBV_ were both significantly higher in the scenario with the short interval (scenario L) than in the scenario with the medium interval (scenario E), both for cows (see Table 2 and Figure 2) and for sires and bulls (see Table 3 and Figure 3). For cows, ∆*F*_ROH_/REF reached 2.06 and 1.47 for the short- and medium-interval scenarios, respectively, while for sires and bulls, this value reached 4.25 and 2.31, respectively. Values of ∆*G*_TBV_/REF were slightly lower: for cows, 1.47 and 1.29 for the short- and medium-interval scenarios, respectively (see Table 2), and for sires and bulls, 1.44 and 1.27, respectively (see Table 3). We observed similar results when we performed the same comparison using high-intensity OPU-IVF-like scenarios (medium generation intervals: scenario J, short generation intervals: scenario M; see Table 2 and Table 3). As with MOET, simulations of OPU-IVF had higher inbreeding rates and increased genetic gain per generation in scenarios with short generation intervals compared to those with medium generation intervals (scenarios E, J, L, and M). The proportion of female donors that were born from female donors was higher for scenarios with short generation intervals than for those with medium generation intervals (see Supplementary Figure S1).

#### 3.3.2. Type of RT Used

We then evaluated high-intensity scenarios to determine the impact of the type of RT used: MOET versus OPU-IVF. Specifically, we compared the effect on ∆*F*_ROH_ and ∆*G*_TBV_ of differences in the number of distinct sires used to generate 15 calves (five for MOET versus 15 for OPU-IVF). For high-intensity scenarios with a medium generation interval, ∆*F*_ROH_ and ∆*G*_TBV_ were not significantly different between the MOET-like (scenario E) and the OPU-IVF-like (scenario J) scenarios for sires and bulls (see Table 3 and Figure 3). For cows, though, ∆*F*_ROH_ was significantly higher and ∆*G*_TBV_ significantly lower for the MOET-like scenario (∆*F*_ROH_/REF = 1.47, ∆*G*_TBV_/REF = 1.29) than for the OPU-IVF-like scenario (∆*F*_ROH_/REF = 1.42, ∆*G*_TBV_/REF = 1.30; see Table 2 and Figure 2). However, the difference between the two scenarios was small for both values, with an increase of ∆*F*_ROH_/REF by a factor of 1.04 (=1.47/1.42) for the OPU-IVF-like scenario with respect to the MOET-like scenario, and an increase of ∆*G*_TBV_/REF by a factor of 1.01 (=1.30/1.29) for the MOET-like scenario compared to the OPU-IVF-like scenario. For high-intensity scenarios with a short generation interval, we observed only one small but significant difference in ∆*G*_TBV_ for cows: ∆*G*_TBV_/REF was 1.47 in the MOET-like scenario (scenario L) and 1.48 in the OPU-IVF-like scenario (scenario M; see Table 2 and Figure 2).

### 3.4. Number of Sires of Bulls and of Marketed Bulls

We compared the impact of the number of sires of bulls and of marketed bulls on ∆*F*_ROH_ and ∆*G*_TBV_ in the context of high-intensity MOET-like scenarios with 150 female donors and medium generation intervals.

Two types of comparisons were performed. First, we compared the impact of a reduction in the number of sires of bulls while keeping constant the number of marketed bulls. Second, we compared the impact of a reduction in the number of marketed bulls while keeping constant the number of sires of bulls.

#### 3.4.1. Number of Sires of Bulls

With the number of marketed bulls held constant, a reduction in the number of sires of bulls resulted in significantly lower values of ∆*G*_TBV_, both for cows and for sires and bulls. A reduction from 80 (scenario E) to 60 (scenario G) sires of bulls, with 60 marketed bulls, resulted in a decrease in ∆*G*_TBV_/REF from 1.29 to 1.25 for cows and from 1.27 to 1.23 for sires and bulls. A reduction from 60 (scenario H) to 40 (scenario I) sires of bulls, with 40 marketed bulls, resulted in a decrease of ∆*G*_TBV_/REF from 1.31 to 1.22 for cows (see Table 2 and Figure 2) and from 1.29 to 1.21 for sires and bulls (see Table 3 and Figure 3). No clear trend was observed for ∆*F*_ROH_: differences between scenarios/populations were significant or not, and changed in direction, depending on the number of marketed bulls and on the population under consideration.

#### 3.4.2. Number of Marketed Bulls

With the number of sires of bulls held constant, a reduction in the number of marketed bulls resulted in significantly higher values of ∆*G*_TBV_, both for cows and for sires and bulls, and significantly higher values of ∆*F*_ROH_ for cows only. A reduction from 60 (scenario G) to 40 (scenario H) marketed bulls, with 60 sires of bulls, resulted in an increase in ∆*G*_TBV_/REF from 1.25 to 1.31 for cows (see Table 2 and Figure 2) and from 1.23 to 1.29 for sires and bulls (see Table 3 and Figure 3). It also resulted in an increase in ∆*F*_ROH_ from 1.54 to 1.63 for cows (see Table 2 and Figure 2), and from 2.29 to 2.48 for sires and bulls, but this latter change was not significant (see Table 3 and Figure 3).

#### 3.4.3. Effect of RT Type on Reductions in the Number of Sires of Bulls and Marketed Bulls

To further examine the high-intensity scenarios, we next evaluated the differences between the two types of RT (MOET with medium generation interval (E and I) and OPU-IVF with short generation interval (M and N)) in two extreme cases: scenarios with a high number of sires of bulls and marketed bulls (E and M) versus those with a low number of sires and bulls (I and N). Two types of comparisons were performed. First, we compared the impact of the type of RT with the numbers of sires of bulls and marketed bulls held constant; then, we compared the impact of a reduction in the numbers of sires of bulls and marketed bulls within each type of RT.

With the numbers of sires of bulls and marketed bulls held constant, the OPU-IVF-like scenario had significantly higher values of both ∆*F*_ROH_ and ∆*G*_TBV_ than the MOET-like scenario for both cows and sires/bulls (see Table 4).

The impact of RT type was stronger on ∆*F*_ROH_/REF than on ∆*G*_TBV_/REF; the use of OPU-IVF instead of MOET increased ∆*F*_ROH_/REF in cows by a factor of 1.40 (= 2.06/1.47) in the scenario with 80 sires of bulls and 60 marketed bulls and a factor of 1.44 (=2.31/1.60) in the scenario with 40 sires of bulls and 40 marketed bulls. In sires and bulls in those same two scenarios, the use of OPU-IVF instead of MOET increased ∆*F*_ROH_/REF by a factor of 1.87 (= 4.33/2.31) and 1.69 (= 4.67/2.76), respectively. In comparison, ∆*G*_TBV_/REF increased for cows by a factor of 1.15 (= 1.48/1.29) in the scenario with 80 sires of bulls and 60 marketed bulls and a factor of 1.14 (= 1.39/1.22) in the scenario with 40 sires of bulls and 40 marketed bulls. For sires and bulls in the same two scenarios, the use of OPU-IVF increased ∆*G*_TBV_/REF by a factor of 1.14 (= 1.45/1.27) and 1.12 (= 1.36/1.21), respectively.

For both MOET and OPU-IVF, drastic reductions in the number of sires of bulls and of marketed bulls resulted in a significant increase in ∆*F*_ROH_ for cows only (see Table 4), and significant decreases in ∆*G*_TBV_ for both cows and sires/bulls (see Table 4).

## 4. Discussion

The objective of this study was to quantify the genetic gain and loss of genetic diversity resulting from changes in different parameters of breeding schemes (use of RT and dimensions of breeding schemes), in the context of genomic selection in dairy cattle breeds. We simulated 15 breeding schemes for genomic selection that differed in their dimensions, and the type and intensity of RT used, and we evaluated these scenarios in terms of annual genetic gain and annual ROH-based inbreeding rate. Although the simulations were designed based on breeding schemes used in French dairy cattle breeds, they reflect the breeding schemes currently used in large dairy cattle breeds using RT.

When no RT was used, the annual ROH-based inbreeding rate (∆*F*_ROH_) was 0.095% in cows and 0.047% in sires and bulls (0.235% and 0.094% per generation, respectively). From the tested scenarios, the highest values of ∆*F*_ROH_ for both cows and sires/bulls were observed in the high-intensity OPU-IVF-like scenario with a short generation interval and low numbers of both sires and bulls, with ∆*F*_ROH_ = 0.219% and 0.220%, respectively (0.501% and 0.356% per generation, respectively). These values were close to the real ones observed in France for the Montbéliarde and Normande breeds, for which the annual ROH-based inbreeding rates in sires and bulls born between 2012 and 2015 were 0.076% and 0.14%, respectively [7]. This similarity supports the relevance of our simulations in mimicking the loss of genetic diversity in dairy cattle breeding schemes. In all studied scenarios, the per-generation ROH-based inbreeding rate stayed under the maximum acceptable threshold of 1% (set by FAO (Food and Agriculture Organization) guidelines [40]), probably thanks to the constraints that we implemented in the simulated breeding schemes (i.e., maximum number of calves per bull or per cow that could become candidates for selection). These constraints were based on current practices in large French dairy cattle breeding schemes, specifically those for Montbéliarde and Normande.

The smallest values of ∆*F*_ROH_ and ∆*G*_TBV_ were always observed when no RT was used. As the intensity of RT increased, both ∆*F*_ROH_ and ∆*G*_TBV_ also increased, regardless of the number of female donors used (150 or 300). In addition, increasing the number of female donors from 150 to 300 always resulted in significantly higher ∆*G*_TBV_ regardless of the intensity of RT use. These results were all consistent with the findings of previous studies [9,10,19,20]. Instead, changes in the number of female donors did not seem to have any consistent effect on inbreeding rate, ∆*F*_ROH_; this was also in agreement with a previous report that increasing the intensity of RT use (i.e., the number of calves per female donor) had a greater impact on ∆*F*_ROH_ than increasing the number of female donors [9].

These results can probably be explained by the fact that the intensity of selection was less affected by the number of female donors than by the number of calves per donor. Regardless of the total number of donors (150 or 300), the offspring of the best females were selected first. This means that it was largely the same 150 donors that experienced the most intense selection in each scenario, and thus, total selection intensity might not have changed very much among different scenarios. Instead, an increase in the number of calves born per female intensified the use of the best female donors based on EBV. Therefore, the higher the intensity of RT use, the higher the selection intensity for the top female donors. Here, constraints on the numbers of sons and daughters that could be selected per donor made it possible to limit the selection intensity. Without these constraints, ∆*F*_ROH_ and ∆*G*_TBV_ would probably have been even higher in the scenarios with high intensity RT, with the risk of severe reductions in genetic diversity and, ultimately, too little genetic variability to be able to increase genetic gain in the medium- or long-term [2].

We then explored the differences between MOET and OPU-IVF. OPU-IVF can be performed sooner in the life of the heifer than MOET as it does not require the donor to have matured enough to develop ovulation cycles [25]. The breeding schemes that we simulated included up to five flushings (corresponding to five sessions) of MOET or 15 sessions of OPU-IVF per year. In France, as of 2018, an average of 5.3 viable embryos was retrieved per flushing for MOET, with one bull used per flushing, while an average of 1.95 viable embryos were retrieved per session for OPU-IVF, with one bull per session [41]. In our simulations, success rates of gestation and birth were both set to 40% following both techniques. This meant that, over the course of a year, it would be possible to obtain 15 calves from five different bulls (three calves times five flushings) with MOET, or 15 calves from 15 different sires (one calf times 15 sessions) using OPU-IVF. Over the same amount of time, then, it is possible to obtain calves from more bulls using OPU-IVF than using MOET. We investigated the use of these techniques by simulating high-intensity MOET-like and OPU-IVF-like scenarios using different parameters.

We first estimated the impact of the interval between generations of female donors by comparing high-intensity scenarios that had either medium- or short-generation intervals (calving at 26 months and calving at 14 months, respectively). Unsurprisingly, we observed that a reduction in the generation interval led to significantly higher values of ∆*F*_ROH_ and ∆*G*_TBV_ for both types of RT, as was reported in a previous study [10]. For a given generation interval, we did not observe clear differences between the types of RT in terms of either ∆*F*_ROH_ or ∆*G*_TBV_. Therefore, it seems that the differences between MOET and OPU-IVF were probably mostly due to the reduction in generation interval that the latter method permits. Moreover, reducing the generation interval had a stronger effect on ∆*F*_ROH_ than on ∆*G*_TBV_, which indicates that drastic reductions in generation intervals between female donors could have a strong detrimental effect on genetic diversity for relatively little genetic gain.

When all other factors were held constant, the differences in the numbers of distinct sires of bulls mated with each female donor (higher for OPU-IVF than for MOET) had a low impact on both genetic gain and genetic diversity. In all comparisons, we held constant the total numbers of sires of bulls, female donors, and calves born per female donor, which were all dependent on the size of the breeding scheme. Therefore, the only difference between MOET-like and OPU-IVF-like scenarios, for a given generation interval, was how sires of bulls and female donors were mated. For both methods, all sires of bulls and female donors were used, and the constraints we applied ensured that all sires of bulls and female donors had male calves chosen as candidates for selection and female calves chosen as female donors. Moreover, matings between the female donors and sires of bulls were performed randomly, which probably homogenized the genetic differences between their calves to a certain extent. It is therefore possible that our simulations did not generate a sufficient degree of difference between the MOET-like and OPU-IVF-like scenarios in the genetic quality of calves chosen as candidates for selection or as female donors.

Instead, a reduction in the generation interval between female donors had a much higher impact on the annual change in genetic diversity and genetic gain. Indeed, given a constant per-generation genetic gain or inbreeding rate, a shortening of the generation interval will increase the subsequent annual rates. However, our simulations showed that a reduction in the generation interval (from medium to short) also increased inbreeding rates and genetic gain per generation. Therefore, the impact of the reduced generation interval between female donors was not only due to the subsequent increase in annual inbreeding rates and genetic gain. In short-interval scenarios, the proportion of female donors that were born from female donors was higher than in medium-interval scenarios. Shortening the generation interval thus has two consequences that combine to increase the genetic gain and the inbreeding rate: (i) a multiplier effect on the annual inbreeding rate and genetic gain and (ii) an increase in the proportion of female donors born from female donors and the overuse of a subset of female donors.

Taken together, our results suggest that the use of OPU-IVF instead of MOET to reduce the generation interval between female donors might lead to detrimental effects on genetic diversity for little genetic gain. OPU-IVF is more invasive than MOET, as it first requires epidural anesthesia and then oocyte aspiration by needle [42,43]. This raises questions about the impact of ovum pick-up on the welfare of heifers, especially after repeated samplings [44]. Consequently, considering its adverse effects on both genetic diversity and animal welfare, OPU-IVF is probably not the optimal RT for dairy cattle breeding schemes.

For both types of RT, a reduction in the number of sires of bulls and marketed bulls resulted in a significant decrease in ∆*G*_TBV_ and a significant increase in ∆*F*_ROH_. Regardless of the numbers of sires of bulls and marketed bulls used in the breeding scheme, the impact of using OPU-IVF rather than MOET was more deleterious to ∆*F*_ROH_ than it was beneficial to ∆*G*_TBV_, even though both values increased significantly. It had previously been predicted that increasing the numbers of sires and bulls in a breeding scheme could alleviate the impact of RT on genetic diversity without seriously compromising genetic gain [10]. However, this approach would increase the costs of breeding schemes, which breeding companies might find unacceptable. In this context, maintaining the number of sires and bulls at least at current levels would keep costs constant, for a higher genetic gain than would be achieved with a lower (as shown in this study) or higher [10] number of sires and bulls. This would not decrease the loss of genetic diversity, but would at least avoid its acceleration.

The costs of the different scenarios were not taken into account in our simulations as the objective of this study did not include an evaluation of the financial aspects of RT. However, for breeding companies, the more intensive the use of RT and the higher the numbers of female donors, the more expensive the breeding of bulls will be. These increased costs could require a corresponding increase in the price of semen doses. This increase in price would be acceptable only if the use of RT yielded bulls of significantly higher genetic quality. The balance between the costs and benefits of RT will vary among species and breeds as well as based on the breeding goals and the monetary value of the trait under selection. In addition, the overall organization of selection and reproduction processes (e.g., use of artificial insemination, veterinary support, etc.) could also play a role. For example, a study by Granleese et al. (2019) [19] of Australian sheep showed that for two different breeding objectives, the genetic gain enabled by the use of RT had different costs and benefits for different kinds of breeders. Therefore, the profitability of RT use for breeding companies and breeders depends on many parameters. Decisions on the use of RT must be made on a case-by-case basis, after careful consideration of the costs and benefits generated according to the breeding objective and breed.

In our simulations, intensification of RT use combined with a reduction in the number of sires and bulls had a detrimental impact on genetic diversity by increasing inbreeding rates. For a given genetic gain, the scenarios that best maintained genetic diversity were those with a medium intensity of RT use or those with a higher number of female donors to compensate for the increase in RT intensity.

Our results were obtained from simulations of a breeding scheme that used random mating and that imposed constraints on the number of calves per bull and female donor that could become bulls or donors themselves. With this design, we might have overestimated genetic gains and inbreeding rates in comparison with breeding schemes that use avoidance mating [45] or optimal contributions (OC) [19,46,47]. The recommendations provided by OC algorithms can be quite useful, as this technique aims to produce a list of individuals for breeding in which global relatedness is minimized. However, any substitutions in the breeding population can completely change the expected overall relatedness, which can limit the practical application of this approach. Although changes in the mating strategy could certainly affect the values obtained by our simulations, such modifications would affect all of the studied scenarios equally, and we would thus not expect them to change the outcomes of any of the comparisons performed here.

Inbreeding depression depends on both the inbreeding rate and the inbreeding load (i.e., the set of deleterious variants carried by the population). In future studies, it would be interesting to identify the genomic regions carrying these variants and to incorporate this information into genomic evaluations. With this knowledge, genomic selection programs would be able to focus more on maintaining genetic diversity in the regions where it is truly important. Such a strategy could limit the impact of the loss of genetic diversity on the health and performance of dairy cattle breeds even with an intensification in the use of RT.

## 5. Conclusions

Our simulations predicted that intensive use of reproductive technologies could lead to improved genetic gain, but this would be accompanied by a deterioration in genetic diversity. The larger genetic gain that we found in scenarios based on OPU-IVF compared to MOET appeared to be, in large part, the result of the significant reduction in the generation interval enabled by OPU-IVF. However, this shortened generation interval led to significant increases in the inbreeding rate, suggesting that using RT to drastically reduce the generation intervals between female donors could have severe detrimental effects on genetic diversity in dairy cattle breeds. In addition, reducing the number of sires of bulls and marketed bulls in the breeding scheme had a detrimental impact on both genetic gain and genetic diversity.

These results led us to the conclusion that two of the major trends in dairy cattle breeding—the intensified use of RT and the cost-conscious reduction in the number of sires of bulls and marketed bulls used by breeding companies—pose serious risks to the genetic diversity of these breeds. In the context of genomic selection and according to the level of genetic gain breeding companies want to reach, avoiding OPU-IVF in favor of MOET, limiting the intensity of use of MOET while maintaining the number of sires and bulls are good practices to maintain genetic diversity. It is also possible to compensate (to a certain point) for an increase in the intensity of use of MOET by increasing the number of female donors and putting constraints on the number of bulls having the same mother, while still maintaining the number of sires and bulls.

It is necessary to find solutions to the loss of genetic diversity, or, at a minimum, approaches that can mitigate the consequences of this loss (i.e., inbreeding depression). One approach that might be effective would involve managing genetic diversity at the genome level by locating the genetic load of deleterious mutations and focusing on the diversity of these regions in particular.

## Figures and Tables

**Table 1:** Parameters used in different scenarios of the simulated dairy cattle breeding program based on the use of embryo transfer.

Scenario		Total Number of Calves Born per Female Donor	Age of Female Donors at Birth of Their Embryo-Transfer Calves	Number of Distinct Sires of Bulls Mated with Each Female Donor	Number of Female Donors (Embryos or Oocytes)	Number of Sires of Bulls	Number of Marketed Bulls
REF	No use of RT	/	/	/	0	80	60
A	Low intensity	3 (1 flushing)	26 months	1 (1 per flushing)	150	80	60
B	Low intensity 300	3 (1 flushing)	26 months	1 (1 per flushing)	300	80	60
C	Medium intensity	9 (3 flushings)	26 months	3 (1 per flushing)	150	80	60
D	Medium intensity 300	9 (3 flushings)	26 months	3 (1 per flushing)	300	80	60
E	High intensity MOET-like	15 (5 flushings)	26 months	5 (1 per flushing)	150	80	60
F	High intensity 300 MOET-like	15 (5 flushings)	26 months	5 (1 per flushing)	300	80	60
G	High intensity MOET-like, medium sires	15 (5 flushings)	26 months	5 (1 per flushing)	150	60	60
H	High intensity MOET-like, medium sires, low bulls	15 (5 flushings)	26 months	5 (1 per flushing)	150	60	40
I	High intensity MOET-like, low sires, low bulls	15 (5 flushings)	26 months	5 (1 per flushing)	150	40	40
J	High intensity OPU-IVF-like	15	26 months	15 (1 per calf)	150	80	60
K	High intensity 300 OPU-IVF-like	15	26 months	15 (1 per calf)	300	80	60
L	High intensity MOET-like, short interval	15 (5 flushings)	14 months	5 (1 per flushing)	150	80	60
M	High intensity OPU-IVF-like, short interval	15	14 months	15 (1 per calf)	150	80	60
N	High intensity OPU-IVF-like, short interval, low sires, low bulls	15	14 months	15 (1 per calf)	150	40	40

RT: Reproductive technologies. MOET: Multiple ovulation and embryo transfer. OPU-IVF: Ovum pick-up and in vitro fertilization.

**Table 2:** Annual runs of homozygosity (ROH)-based inbreeding rate versus annual genetic gain among cows.

Scenario		Annual ROH-Based Inbreeding Rate in % [95% Confidence Interval]	Annual ROH-Based Inbreeding Rate Compared with Scenario REF	Annual Genetic Gain in TBV [95% Confidence Interval]	Annual Genetic Gain in TBV Compared with Scenario REF
REF	No use of RT	0.095 ^a^ [0.094;0.096]	1.00 ^a^	0.298 ^a^ [0.297;0.298]	1.00 ^a^
A	Low intensity	0.100 ^b^ [0.099;0.101]	1.05 ^b^	0.323 ^b^ [0.322;0.323]	1.08 ^b^
B	Low intensity 300	0.103 ^c^ [0.102;0.104]	1.08 ^c^	0.332 ^c^ [0.332;0.333]	1.11 ^c^
C	Medium intensity	0.127 ^d^ [0.126;0.128]	1.34 ^d^	0.364 ^d^ [0.363;0.364]	1.22 ^d^
D	Medium intensity 300	0.124 ^e^ [0.123;0.125]	1.31 ^e^	0.381 ^e^ [0.381;0.382]	1.28 ^e^
E	High intensity MOET-like	0.140 ^f^ [0.139;0.141]	1.47 ^f^	0.384 ^f^ [0.384;0.384]	1.29 ^f^
F	High intensity 300 MOET-like	0.134 ^g^ [0.133;0.135]	1.41 ^g^	0.403 ^g^ [0.403;0.403]	1.35 ^g^
G	High intensity MOET-like, medium sires	0.146 ^h^ [0.145;0.147]	1.54 ^h^	0.373 ^h^ [0.372;0.373]	1.25 ^h^
H	High intensity MOET-like, medium sires, low bulls	0.155 ^i^ [0.154;0.156]	1.63 ^i^	0.389 ^i^ [0.389;0.389]	1.31 ^i^
I	High intensity MOET-like, low sires, low bulls	0.152 ^j^ [0.151;0.153]	1.60 ^j^	0.365 ^j^ [0.365;0.366]	1.22 ^j^
J	High intensity OPU-IVF-like	0.135 ^g^ [0.134;0.136]	1.42 ^g^	0.386 ^k^ [0.386;0.386]	1.30 ^k^
K	High intensity 300 OPU-IVF-like	0.146 ^h^ [0.145;0.147]	1.54 ^h^	0.407 ^l^ [0.407;0.407]	1.37 ^l^
L	High intensity MOET-like, short interval	0.196 ^k^ [0.195;0.197]	2.06 ^k^	0.438 ^m^ [0.438;0.439]	1.47 ^m^
M	High intensity OPU-IVF-like, short interval	0.196 ^k^ [0.195;0.197]	2.06 ^k^	0.440 ^n^ [0.440;0.440]	1.48 ^n^
N	High intensity OPU-IVF-like, short interval, low sires, low bulls	0.219 ^l^ [0.218;0.220]	2.31 ^l^	0.415 ^o^ [0.415;0.416]	1.39 ^o^

Comparisons with REF are calculated as the ratio between the estimated value of the parameter for a given scenario and the estimated value of the parameter for the reference scenario REF (no use of embryo transfer). RT: Reproductive technologies. MOET: Multiple ovulation and embryo transfer. OPU-IVF: Ovum pick-up and in vitro fertilization. ROH: Runs of Homozygosity. TBV: True Breeding Value. ^a–o^ Within each population (cows or sires and bulls), values within a column with different superscripts are significantly different (*p*-value < 0.05).

**Table 3:** Annual ROH-based inbreeding rate versus annual genetic gain among sires and bulls.

Scenario		Annual ROH-based Inbreeding Rate in % [95% Confidence Interval]	Annual ROH-based Inbreeding Rate Compared with Scenario REF	Annual Genetic Gain in TBV [95% Confidence Interval]	Annual Genetic Gain in TBV Compared with Scenario REF
REF	No use of RT	0.04 ^a^ [0.038;0.056]	1.00 ^a^	0.280 ^a^ [0.277;0.282]	1.00 ^a^
A	Low intensity	0.050 ^a^ [0.041;0.058]	1.05 ^a^	0.303 ^b^ [0.301;0.305]	1.08 ^b^
B	Low intensity 300	0.057 ^ab^ [0.048;0.066]	1.21 ^ab^	0.314 ^c^ [0.312;0.316]	1.12 ^c^
C	Medium intensity	0.076 ^b^ [0.067;0.084]	1.60 ^b^	0.340 ^d^ [0.338;0.342]	1.21 ^d^
D	Medium intensity 300	0.078 ^b^ [0.069;0.086]	1.65 ^b^	0.357 ^e^ [0.355;0.359]	1.28 ^e^
E	High intensity MOET-like	0.109 ^c^ [0.100;0.117]	2.31 ^c^	0.356 ^e^ [0.354;0.359]	1.27 ^e^
F	High intensity 300 MOET-like	0.109 ^c^ [0.100;0.117]	2.31 ^c^	0.374 ^f^ [0.372;0.376]	1.34 ^f^
G	High intensity MOET-like, medium sires	0.108 ^c^ [0.098;0.118]	2.29 ^c^	0.344 ^d^ [0.342;0.347]	1.23 ^d^
H	High intensity MOET-like, medium sires, low bulls	0.117 ^c^ [0.107;0.127]	2.48 ^c^	0.361 ^e^ [0.359;0.364]	1.29 ^e^
I	High intensity MOET-like, low sires, low bulls	0.130 ^c^ [0.118;0.142]	2.76 ^c^	0.340 ^d^ [0.337;0.343]	1.21 ^d^
J	High intensity OPU-IVF-like	0.112 ^c^ [0.103;0.120]	2.38 ^c^	0.359 ^e^ [0.357;0.361]	1.28 ^e^
K	High intensity 300 OPU-IVF-like	0.116 ^c^ [0.107;0.125]	2.46 ^c^	0.380 ^g^ [0.378;0.382]	1.36 ^g^
L	High intensity MOET-like, short interval	0.200 ^d^ [0.191;0.209]	4.25 ^d^	0.404 ^h^ [0.402;0.406]	1.44 ^h^
M	High intensity OPU-IVF-like, short interval	0.204 ^d^ [0.195;0.212]	4.33 ^d^	0.405 ^h^ [0.403;0.407]	1.45 ^h^
N	High intensity OPU-IVF-like, short interval, low sires, low bulls	0.220 ^d^ [0.208;0.232]	4.67 ^d^	0.380 ^fg^ [0.377;0.383]	1.36 ^fg^

Comparisons with REF are calculated as the ratio between the estimated value of the parameter for a given scenario and the estimated value of the parameter for the reference. scenario REF (no use of embryo transfer). RT: Reproductive technologies. MOET: Multiple ovulation and embryo transfer. OPU-IVF: Ovum pick-up and in vitro fertilization. ROH: Runs of Homozygosity. TBV: True Breeding Value. ^a–h^ Within each population (cows or sires and bulls), values within a column with different superscripts are significantly different (*p*-value < 0.05).

**Table 4:** Annual ROH-based inbreeding rate versus annual genetic gain among cows and sires/bulls for four different scenarios.

**Number of Sires and Bulls**	**Annual ROH-Based Inbreeding Rate Compared with Scenario REF**		**Annual Genetic Gain in TBV Compared with Scenario REF**	
	MOET, Medium Interval	OPU-IVF, Short Interval	MOET, Medium Interval	OPU-IVF, Short Interval
80 sires of bulls and 60 marketed bulls	**Scenario E** 1.47 for cows 2.31 for sires/bulls	**Scenario M** 2.06 for cows 4.33 for sires/bulls	**Scenario E** 1.29 for cows 1.27 for sires/bulls	**Scenario M** 1.48 for cows 1.45 for sires/bulls
40 sires of bulls and 40 marketed bulls	**Scenario I** 1.60 for cows 2.76 for sires/bulls	**Scenario N** 2.31 for cows 4.67 for sires/bulls	**Scenario I** 1.22 for cows 1.21 for sires/bulls	**Scenario N** 1.39 for cows 1.36 for sires/bulls

Comparisons with REF were calculated as the ratio between the estimated value of the parameter for a given scenario and the estimated value of the parameter for the reference scenario REF (no use of embryo transfer). RT: Reproductive technologies. MOET: Multiple ovulation and embryo transfer. OPU-IVF: Ovum pick-up and in vitro fertilization. ROH: Runs of Homozygosity. TBV: True Breeding Value.

## Supplementary Materials

The following are available online at https://www.mdpi.com/2076-2615/10/10/1903/s1, Table S1: Positions and effects of additive quantitative trait loci (QTLs) along the simulated genome used as the basis for simulations of an embryo transfer breeding scheme and variations thereof in French dairy cattle breeds, Table S2: ROH-based inbreeding rates per generation and genetic gains in true breeding value per generation for each scenario of the simulated embryo transfer breeding program in French dairy cattle, Figure S1: Proportion of female donors and of sires and bulls born from embryo transfer over all birth years and replicates for each scenario of the simulated embryo transfer breeding scheme in French dairy cattle breeds.

## References

[1] Stachowicz K., Sargolzaei M., Miglior F., Schenkel F.S. (2011). Rates of inbreeding and genetic diversity in Canadian Holstein and Jersey cattle. J. Dairy Sci..

[2] Dickerson G.E., Hazel L.N. (1944). Effectiveness of selection on progeny performance as a supplement to earlier culling in livestock. J. Agric. Res..

[3] Notter D.R. (1999). The importance of genetic diversity in livestock populations of the future. J. Anim. Sci..

[4] Leroy G. (2014). Inbreeding depression in livestock species: Review and meta-analysis. Anim. Genet..

[5] Pryce J.E., Haile-Mariam M., Goddard M.E., Hayes B.J. (2014). Identification of genomic regions associated with inbreeding depression in Holstein and Jersey dairy cattle. Genet. Sel. Evol..

[6] Eynard S.E., Windig J.J., Hiemstra S.J., Calus M.P.L. (2016). Whole-genome sequence data uncover loss of genetic diversity due to selection. Genet. Sel. Evol..

[7] Doublet A.-C., Croiseau P., Fritz S., Michenet A., Hozé C., Danchin-Burge C., Laloë D., Restoux G. (2019). The impact of genomic selection on genetic diversity and genetic gain in three French dairy cattle breeds. Genet. Sel. Evol..

[8] Weigel K.A. (2001). Controlling Inbreeding in Modern Breeding Programs. J. Dairy Sci..

[9] Bouquet A., Sørensen A.C., Juga J. (2015). Genomic selection strategies to optimize the use of multiple ovulation and embryo transfer schemes in dairy cattle breeding programs. Livest. Sci..

[10] Thomasen J.R., Willam A., Egger-Danner C., Sørensen A.C. (2016). Reproductive technologies combine well with genomic selection in dairy breeding programs. J. Dairy Sci..

[11] Maignel L., Boichard D., Verrier E. (1996). Genetic variability of French dairy breeds estimated from pedigree information. Interbull Bull..

[12] Le Mézec P., Danchin-Burge C., Moureaux S. Davantage de diversité avec la génomique? plutôt non.... http://idele.fr/contact/publication/idelesolr/recommends/les-programmes-de-selection-et-de-diffusion-de-taureaux-dia-a-lere-de-la-genomique-et-leurs-effets.html.

[13] Danchin-Burge C., Danvy S., Laloë D., Verrier E. (2017). Création d’un observatoire de la VARiabilité génétique des RUMinants et des Equidés (VARUME). Innov. Agron..

[14] Eynard S.E., Windig J.J., Leroy G., van Binsbergen R., Calus M. (2015). The effect of rare alleles on estimated genomic relationships from whole genome sequence data. BMC Genet..

[15] Thomasen J.R., Liu H., Sørensen A.C. (2020). Genotyping more cows increases genetic gain and reduces rate of true inbreeding in a dairy cattle breeding scheme using female reproductive technologies. J. Dairy Sci..

[16] Kardos M., Luikart G., Allendorf F.W. (2015). Measuring individual inbreeding in the age of genomics: Marker-based measures are better than pedigrees. Heredity.

[17] Doekes H.P., Veerkamp R.F., Bijma P., Hiemstra S.J., Windig J.J. (2018). Trends in genome-wide and region-specific genetic diversity in the Dutch-Flemish Holstein–Friesian breeding program from 1986 to 2015. Genet. Sel. Evol..

[18] Forutan M., Ansari Mahyari S., Baes C., Melzer N., Schenkel F.S., Sargolzaei M. (2018). Inbreeding and runs of homozygosity before and after genomic selection in North American Holstein cattle. BMC Genom..

[19] Granleese T., Clark S.A., Kinghorn B.P., Werf J.H.J. (2019). van der Optimizing female allocation to reproductive technologies considering merit, inbreeding and cost in nucleus breeding programmes with genomic selection. J. Anim. Breed. Genet..

[20] Pedersen L.D., Kargo M., Berg P., Voergaard J., Buch L.H., Sørensen A.C. (2012). Genomic selection strategies in dairy cattle breeding programmes: Sexed semen cannot replace multiple ovulation and embryo transfer as superior reproductive technology. J. Anim. Breed. Genet..

[21] Pryce J.E., Goddard M.E., Raadsma H.W., Hayes B.J. (2010). Deterministic models of breeding scheme designs that incorporate genomic selection. J. Dairy Sci..

[22] Sørensen A.C., Sørensen M.K. Inbreeding Rates in Breeding Programs with Different Strategies for Using Genomic Selection. Proceedings of the 2009 Interbull Meeting.

[23] Thomasen J.R., Egger-Danner C., Willam A., Guldbrandtsen B., Lund M.S., Sørensen A.C. (2014). Genomic selection strategies in a small dairy cattle population evaluated for genetic gain and profit. J. Dairy Sci..

[24] Land R.B., Hill W.G. (1975). The possible use of superovulation and embryo transfer in cattle to increase response to selection. Anim. Sci..

[25] Galli C., Crotti G., Notari C., Turini P., Duchi R., Lazzari G. (2001). Embryo production by ovum pick up from live donors. Theriogenology.

[26] Pook T. (2018). MoBPS: Simulation of Breeding Programs.

[27] Pook T., Schlather M., Simianer H. (2020). MoBPS-Modular Breeding Program Simulator. G3 Genes Genomes Genet..

[28] Arias J.A., Keehan M., Fisher P., Coppieters W., Spelman R. (2009). A high density linkage map of the bovine genome. BMC Genet..

[29] Hayes B., Goddard M. (2001). The distribution of the effects of genes affecting quantitative traits in livestock. Genet. Sel. Evol..

[30] Eynard S.E., Croiseau P., Laloë D., Fritz S., Calus M.P.L., Restoux G. (2018). Which Individuals to Choose to Update the Reference Population? Minimizing the Loss of Genetic Diversity in Animal Genomic Selection Programs. G3 Bethesda Md.

[31] Laloë D., Phocas F. (2003). A proposal of criteria of robustness analysis in genetic evaluation. Livest. Prod. Sci..

[32] Mardia K.V., Kent J.T., Bibby J.M. (1979). Multivariate Analysis.

[33] McQuillan R., Leutenegger A.-L., Abdel-Rahman R., Franklin C.S., Pericic M., Barac-Lauc L., Smolej-Narancic N., Janicijevic B., Polasek O., Tenesa A. (2008). Runs of Homozygosity in European Populations. Am. J. Hum. Genet..

[34] de Cara M.Á.R., Villanueva B., Toro M.Á., Fernández J. (2013). Using genomic tools to maintain diversity and fitness in conservation programmes. Mol. Ecol..

[35] Chang C.C., Chow C.C., Tellier L.C., Vattikuti S., Purcell S.M., Lee J.J. (2015). Second-generation PLINK: Rising to the challenge of larger and richer datasets. GigaScience.

[36] Purcell S., Chang C. PLINK 1.9.

[37] R Core Team (2019). R: A Language and Environment for Statistical Computing.

[38] Lenth R., Singmann H., Love J., Buerkner P., Herve M. (2020). emmeans: Estimated Marginal Means, aka Least-Squares Means.

[39] Hothorn T., Bretz F., Westfall P., Heiberger R.M., Schuetzenmeister A., Scheibe S. (2020). multcomp: Simultaneous Inference in General Parametric Models.

[40] FAO (2000). Secondary Guidelines for the National Farm Animal Genetic Resources Management Plans: Management of Small Populations at Risk.

[41] AETE (2019). Commercial Embryo Transfer Activity in Europe 2018.

[42] Petyim S., Bage R., Forsberg M., Rodriguez-Martinez H., Larsson B. (2000). The Effect of Repeated Follicular Puncture on Ovarian Function in Dairy Heifers. J. Vet. Med. Ser. A.

[43] Qi M., Yao Y., Ma H., Wang J., Zhao X., Liu L., Tang X., Zhang L., Zhang S., Sun F. (2013). Transvaginal Ultrasound-guided Ovum Pick-up(OPU) in Cattle. J. Biomim. Biomater. Tissue Eng..

[44] Petyim S., Båge R., Madej A., Larsson B. (2007). Ovum Pick-up in Dairy Heifers: Does it Affect Animal Well-being?. Reprod. Domest. Anim..

[45] Bérodier M., Berg P., Meuwissen T., Brochard M., Ducrocq V. Improving mating plans at herd level using genomic information. Proceedings of the Annual Meeting of the European Association for Animal Production (EAAP).

[46] Meuwissen T.H.E. (1997). Maximizing the response of selection with a predefined rate of inbreeding. J. Anim. Sci..

[47] Granleese T., Clark S.A., Swan A.A., van der Werf J.H.J. (2015). Increased genetic gains in sheep, beef and dairy breeding programs from using female reproductive technologies combined with optimal contribution selection and genomic breeding values. Genet. Sel. Evol..

