# Supplementary material for: Intensified Use of Reproductive Technologies and Reduced Dimensions of Breeding Schemes Put Genetic Diversity at Risk in Dairy Cattle Breeds
Source: Animals (Basel). 2020 Oct 17;10(10):1903. doi: 10.3390/ani10101903 (PMC7650664; doi:10.3390/ani10101903)
Supplement: Supplementary file 1 [file animals-10-01903-s001.zip › Suppl_mat_Table_S2.docx]

**Table S2.** ROH-based inbreeding rates per generation and genetic gains in true breeding value per generation for each scenario of the simulated embryo transfer breeding program in French dairy cattle.

| **Population** | **Scenario** | **ROH-based inbreeding rate per generation in %** | **ROH-based inbreeding rate per generation compared with scenario REF** | **Genetic gain in TBV per generation** | **Genetic gain in TBV per generation compared with scenario REF** |
| --- | --- | --- | --- | --- | --- |
| Cows | REF | 0.235 | 1.00 | 0.738 | 1.00 |
|  | A | 0.245 | 1.04 | 0.793 | 1.07 |
|  | B | 0.253 | 1.07 | 0.814 | 1.10 |
|  | C | 0.308 | 1.31 | 0.882 | 1.20 |
|  | D | 0.295 | 1.25 | 0.907 | 1.23 |
|  | E | 0.334 | 1.42 | 0.917 | 1.24 |
|  | F | 0.312 | 1.32 | 0.937 | 1.27 |
|  | G | 0.349 | 1.48 | 0.89 | 1.21 |
|  | H | 0.370 | 1.57 | 0.929 | 1.26 |
|  | I | 0.363 | 1.54 | 0.872 | 1.18 |
|  | J | 0.322 | 1.37 | 0.922 | 1.25 |
|  | K | 0.340 | 1.44 | 0.947 | 1.28 |
|  | L | 0.448 | 1.90 | 1.001 | 1.36 |
|  | M | 0.448 | 1.90 | 1.005 | 1.36 |
|  | N | 0.501 | 2.13 | 0.949 | 1.28 |
| Sires and bulls | REF | 0.094 | 1.00 | 0.560 | 1.00 |
|  | A | 0.099 | 1.05 | 0.606 | 1.08 |
|  | B | 0.114 | 1.21 | 0.628 | 1.12 |
|  | C | 0.151 | 1.60 | 0.680 | 1.21 |
|  | D | 0.155 | 1.65 | 0.714 | 1.28 |
|  | E | 0.218 | 2.31 | 0.712 | 1.27 |
|  | F | 0.218 | 2.31 | 0.748 | 1.34 |
|  | G | 0.216 | 2.29 | 0.688 | 1.23 |
|  | H | 0.234 | 2.48 | 0.722 | 1.29 |
|  | I | 0.260 | 2.76 | 0.680 | 1.21 |
|  | J | 0.224 | 2.38 | 0.718 | 1.28 |
|  | K | 0.232 | 2.46 | 0.760 | 1.36 |
|  | L | 0.323 | 3.43 | 0.653 | 1.17 |
|  | M | 0.329 | 3.50 | 0.654 | 1.17 |
|  | N | 0.356 | 3.78 | 0.615 | 1.10 |

Comparisons with REF are calculated as the ratio between the estimated value of the parameter for a given scenario and the estimated value of the parameter for the reference scenario (no use of embryo transfer).

ROH: Runs of Homozygosity

TBV: True Breeding Value
