# Supplementary figures and images for: Intensified Use of Reproductive Technologies and Reduced Dimensions of Breeding Schemes Put Genetic Diversity at Risk in Dairy Cattle Breeds
Source: Animals (Basel). 2020 Oct 17;10(10):1903. doi: 10.3390/ani10101903 (PMC7650664; doi:10.3390/ani10101903)

Proportion of female donors and of sires and bulls born from embryo transfer

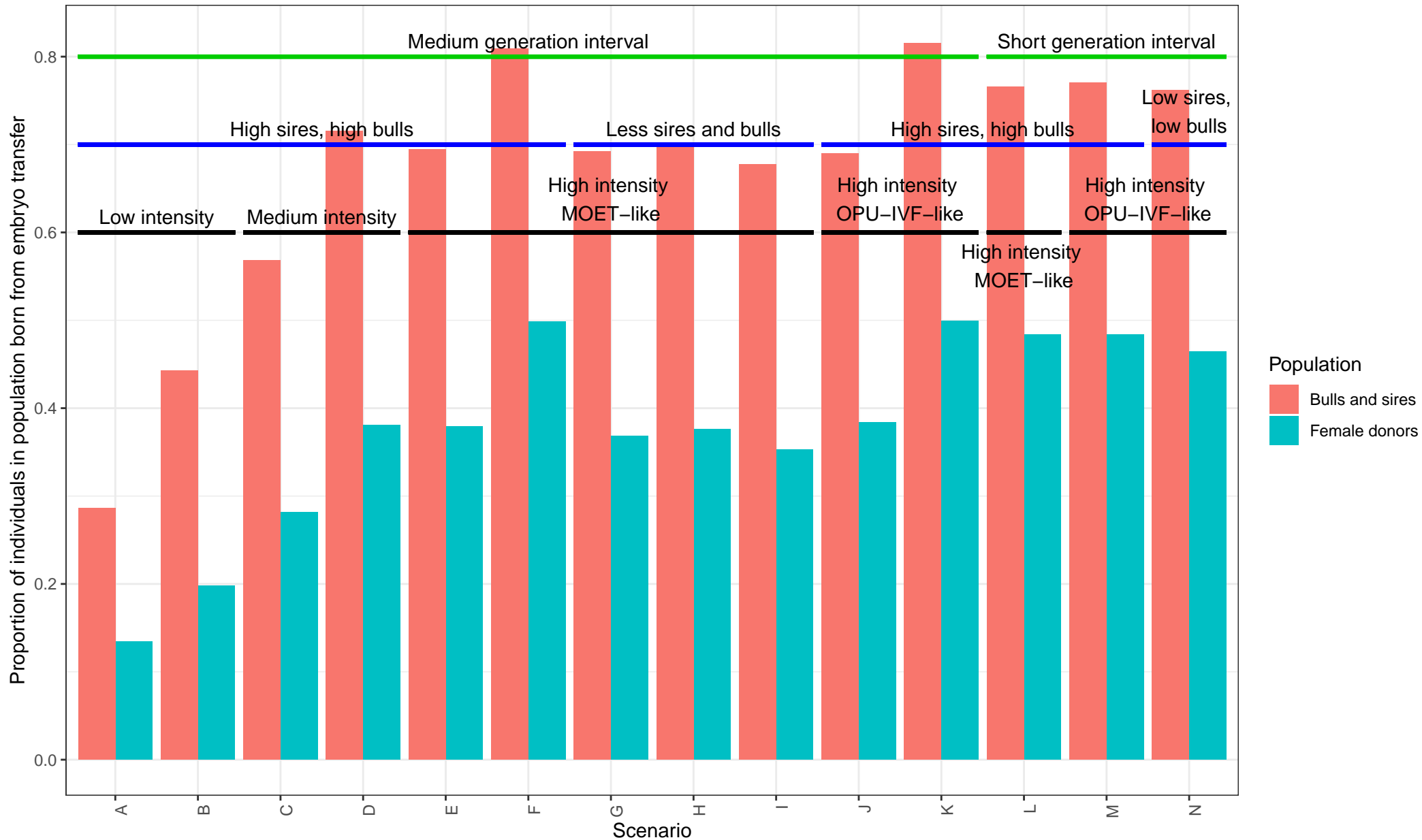

Supplement: Supplementary file 1 [file animals-10-01903-s001.zip › Suppl_mat_Figure_S1.pdf]
